# Mannitol-negative methicillin-resistant *Staphylococcus aureus* from nasal swab specimens in Brazil

**DOI:** 10.1590/S1517-838246220140179

**Published:** 2015-06-01

**Authors:** Danielle Caldeira Martins dos Santos, Thaina Miranda da Costa, Renata Fernandes Rabello, Fábio Aguiar Alves, Silvia Susana Bona de Mondino

**Affiliations:** 1 Universidade Federal Fluminense, Programa de Pós-gradução em Patologia, Faculdade de Medicina, Universidade Federal Fluminense, Niterói, RJ, Brasil, Programa de Pós-gradução em Patologia, Faculdade de Medicina, Universidade Federal Fluminense, Niterói, RJ, Brazil.; 2 Universidade Federal Fluminense, Departamento de Microbiologia e Parasitologia, Instituto Biomédico, Universidade Federal Fluminense, Niterói, RJ, Brasil, Departamento de Microbiologia e Parasitologia, Instituto Biomédico, Universidade Federal Fluminense, Niterói, RJ, Brazil.

**Keywords:** mannitol-negative, MRSA, nasal swab surveillance, SCC*mec*

## Abstract

The isolation of mannitol-negative methicillin-resistant *Staphylococcus aureus* from nasal swabs is reported. Among the 59 isolates, 9 (15%) isolates were mannitol-negative; all of these isolates were categorized as staphylococcal cassette chromosome *mec* (SCC*mec)* type IVa. This report emphasizes that mannitol fermentation on mannitol salt agar should not be used as the sole criterion when screening nasal swab specimens for *S. aureus*.

Methicillin-resistant *Staphylococcus aureus* (MRSA) remains an important cause of nosocomial infections worldwide, with severe consequences for patients and significant healthcare costs. Active surveillance and correct identification of patients colonized with MRSA is an effective infection control strategy that helps prevent the spread of this organism within the hospital environment (Muto *et al.*, 2003). Mannitol salt agar (MSA) is a conventional medium that is frequently used to screen nasal swab specimens for presumptive MRSA colonization based on the growth of yellow colonies derived from the fermentation of mannitol (Chapman, 1945). However, clinical isolates of mannitol-negative MRSA have been widely reported (Kateete *et al.*, 2010; Shittu *et al.*, 2007). The aim of this study was to describe the occurrence of *S. aureus* strains that do not ferment mannitol on MSA, which is an important screening method for the detection of MRSA colonization.

From August 2009 to August 2011 a total of 104 isolates presumed to be *S. aureus* were obtained via nasal swab surveillance of patients admitted to one public hospital and one private hospital located in the city of Niterói in Rio de Janeiro, Brazil. The strains were sub-cultured on MSA medium (Becton, Dickinson and Company, Sparks, Nevada, USA) and incubated at 35 °C for 24 h to confirm mannitol fermentation. Single colonies of Gram-positive, catalase-positive, mannitol-positive or-negative cocci were subjected to the tube coagulase and DNase tests. The tube coagulase test was performed using rabbit plasma (Laborclin, Curitiba, Paraná, Brazil). The DNase test was performed by incubating the isolates on DNase agar medium (Oxoid, Cambridge, UK). The isolates were tested for susceptibility to oxacillin (1 μg) and cefoxitin (30 μg) (Cecon, São Paulo, São Paulo, Brazil) using the disk diffusion method, according to the guidelines of the Clinical and Laboratory Standards Institute (CLSI, 2012).

DNA extraction was performed as previously described by Pacheco *et al.* (1997). PCR amplification was performed using the primers 5′- GTG AAG ATA TAC CAA GTG ATT -3′ and 5′- ATG CGC TAT AGA TTG AAA GGA T -3′ (Eurofins, Huntsville, Alabama, USA), which are specific for the *mecA* gene, according to the methodology proposed by Zhang *et al.* (2005). PCR to detect the *nuc* gene was performed according to the method described by Brakstad *et al.* (1992), using the primers 5′-GCG TTA ACG GAT GGT GAT GTT-3′ and 5′-CAA AGC GCC ACG TTG TAA AAC AGC-3′ (Eurofins, Huntsville, Alabama, USA). A multiplex-PCR assay was performed to characterize staphylococcal cassette chromosome *mec* (SCC*mec*) types I to V in the MRSA strains according to the method developed by Zhang *et al.* (2005).

Of the 104 strains evaluated, 69 (66%) strains were *S. aureus*, according to the results obtained after PCR amplification of the *nuc* gene, and 35 (34%) strains were organisms other than *S. aureus*. The phenotypic characteristics of the strains are shown in Table 1.

**Table 1:** General phenotypic characteristics of 104 *Staphylococcus* spp. according to *nuc* gene PCR results.

*nuc* PCR	MSA* n (%)		Coagulase n (%)		DNase n (%)	
			
	Positive	Negative	Positive	Negative	Positive	Negative
Positive (*S. aureus*) n = 69	59 (86)	10 (14)	60 (87)	9 (13)	61 (88)	8 (12)
Negative (non-*S. aureus*) n = 35	31 (89)	4 (11)	0	35 (100)	0	35 (100)

* MSA: mannitol salt agar.

Among the 69 *S. aureus* strains, 59 (86%) strains harbored the *mecA* gene; of these MRSA strains, 15% (9/59) were mannitol-negative but coagulase- and DNase-positive.

Of the 59 MRSA isolates examined, 43 (73%) isolates were SCC*mec*-typeable with the primers used in this study. SCC*mec* type II was the most common (49%) type, followed by type IV (37%), including sub-types IVa (*n* = 14), IVb (*n* = 1), and IVc (*n* = 1); type III (7%); type I (5%) and type V (2%). The nine mannitol-negative strains were typed as SCC*mec* IVa.

The sensitivity and specificity of each test was calculated to evaluate the performance of the individual tests in the identification of *S. aureus*. The DNase test was the most sensitive test (88% sensitivity), followed by coagulase and MSA (87% and 86% sensitivity, respectively). The coagulase and DNase tests were the most specific tests (100% specificity). The low specificity of mannitol (11%) for detecting *S. aureus* could be caused by the large number of coagulase-negative staphylococci (CoNS) isolates that were mannitol-positive. Mannitol-negative *S. aureus* strains have also been reported by other authors (Kateete *et al.*, 2010; Shittu *et al.*, 2007). The identification of nine of these strains was correct based on the positivity of the coagulase and DNase tests. However, the isolation of MRSA strains with negative results for the coagulase (Bertrand *et al.*, 2002; Del' Alamo *et al.*, 2007) and DNAse (Rao *et al.*, 2002) tests was described previously.

Comparing the presence of the *mecA* gene with the specificity of the disk diffusion method, 57 of the 59 MRSA isolates exhibited consistency levels of 100% (57/57) and 75% (43/57) for cefoxitin and oxacilin, respectively. The cefoxitin disk test correlates well with *mecA* detection using PCR and also remains a good marker for the detection of methicillin resistance in *S. aureus* (Swenson *et al.*, 2005). However, two of the 59 MRSA isolates were susceptible to both of the antimicrobials tested. This phenotype is known as pre-MRSA, in which the isolate harbors a repressed *mecA* gene; in this case, contact with β-lactam antibiotics could trigger the expression of the PBP2a protein (Berger-Bächi *et al.*, 2002).

Worldwide, most HA-MRSA isolates are classified as types I, II and III; most CA-MRSA isolates belong to types IV and V (Chambers & Deleo, 2009). The present study detected nasal MRSA isolates carrying SCC*mec* types I, II, III, IVa, IVb, IVc and V. The most common SCC*mec* detected was type II; this finding is a significant result that has been reported rarely. Silva-Carvalho *et al.* (2009) verified that SCC*mec* types III and IV were the most frequent among nasal MRSA isolates from two hospitals located in the Rio de Janeiro metropolitan region. Notably, all of the mannitol-negative MRSA isolates were categorized as type IVa.

In our study, we detected 35 CoNS isolates, of which 31 (89%) isolates were *mecA*-positive. Most of these isolates were mannitol-positive (28/31; 90%) but coagulase- and DNase test-negative. CoNS species that produce acid from mannitol have been described previously (Bannerman *et al.*, 2007; Kateete *et al.*, 2010). Carriage of the mecA gene is not exclusive to MRSA and has been reported frequently in several CoNS species isolated from human and animal sources (Mombach Pinheiro Machado *et al.*, 2007; Black *et al.*, 2009). This fact emphasizes the importance of CoNS as reservoirs of the *mecA* gene, which can be transmitted to *S. aureus* (Barbier *et al.*, 2010; Tsubakishita *et al.*, 2010).

According to our results, 9 (15%) of the 59 MRSA strains identified in this study were mannitol-negative. Therefore, these strains would not have been detected using mannitol-positive colony growth on MSA as the sole criterion for MRSA screening. Thus, the inclusion of at least two additional tests, such as the coagulase tube and DNase tests, is needed to improve the identification of *S. aureus* strains and the early detection of patients who are infected or colonized by MRSA.

## References

[B01] Bannerman TL, Peacock SJ, Murray PR, Baron EJ, Jorgensen JH (2007). Staphylococcus, Micrococcus and other catalase-positive
cocci. Manual of Clinical Microbiology.

[B02] Barbier F, Ruppé E, Hernandez D (2010). Methicillin-resistant coagulase-negative staphylococci in the
community: high homology of SCC*mec* IVa between
*Staphylococcus epidermidis* and major clones of
methicillin-resistant *Staphylococcus aureus*. J Infect Dis.

[B03] Berger-Bächi B, Rohrer S (2002). Factors influencing methicillin resistance in
staphylococci. Arch Microbiol.

[B04] Bertrand X, Huguenin Y, Talon D (2002). First report of a catalase-negative methicillin-resistant
*Staphylococcus aureus*. Diag Microbiol Infect Dis.

[B05] Black CC, Solyman SM, Eberlein LC (2009). Identification of a predominant multilocus sequence type,
pulsed-field gel electrophoresis cluster, and novel staphylococcal
chromosomal cassette in clinical isolates of
*mecA*-containing, methicillin-resistant
*Staphylococcus pseudintermedius*. Vet Microbiol.

[B06] Brakstad OG, Aasbakk K, Maeland JA (1992). Detection of *Staphylococcus aureus* by Polymerase
Chain Reaction Amplification of the *nuc*
gene. J Clin Microbiol.

[B07] Chambers HF, DeLeo FR (2009). Waves of resistance: *Staphylococcus aureus* in
the antibiotic era. Nat Rev Microbiol.

[B08] Chapman GH (1945). The significance of sodium chloride in studies of
staphylococci. J Bacteriol.

[B09] CLSI (2012). Performance standards for antimicrobial susceptibility testing.
22th Informational Supplement Update. Clinical and Laboratory Standards (M100-S22).

[B10] Del'Alamo L, d'Azevedo PA, Strob AJ (2007). Na outbreak of catalase-negative methicilin-resistant
*Staphylococcus aureus*. J Hosp Infect.

[B11] Kateete DP, Kimani CN, Katabazi FA (2010). Identification of *Staphylococcus aureus*: DNase
and Mannitol salt agar improve the efficiency of the tube coagulase
test. Annal Clin Microbiol Antimicrob.

[B12] Mombach Pinheiro Machado ABM, Reiter KC, Paiva RM, Barth AL (2007). Distribution of staphylococcal cassete chromosome
*mec* (SCC*mec*) types I, II, III and
coagulase negative staphylococci from patients attending a tertiary hospital
in southern Brazil. J Med Microbiol.

[B13] Muto CA, Jernigan JA, Ostrowsky BE (2003). SHEA Guideline for Preventing Nosocomial Transmission of
Multidrug-Resistant Strains of *Staphylococcus aureus* and
*Enterococcus*. Infect Control Hosp Epidemiol.

[B14] Pacheco AB, Guth BE, Soares KC (1997). Random amplification of polymorphic DNA reveals serotype-specific
clonal clusters among enterotoxigenic *Escherichia coli*
strains isolated from humans. J Clin Microbiol.

[B15] Rao JG, Qamruddin AO, Hassan IA (2002). Cluster of clinical isolates of epidemic methicillin-resistant
*Staphylococcus aureus* (EMRSA) with a negative
deoxyribonuclease (DNase) test - implications for laboratory diagnosis and
infection control. J Hosp Infec.

[B16] Severin JA, Lestari ES, Kuntaman K (2010). Nasal carriage of methicillin-resistant and methicillin-sensitive
strains of *Staphylococcus sciuru* in the Indonesian
population. Antimicron Agents Chemother.

[B17] Silva-Carvalho MC, Bonelli RR, Souza RR (2009). Emergence of mul-tiresistant variants of the community-acquired
methicillin-resistant *Staphylococcus aureus* lineage
ST1-SCC*mec*IV in two hospitals in Rio de Janeiro,
Brazil. Diagn Microbiol Infect Dis.

[B18] Shittu A, Lin J, Morrison D (2007). Molecular identification and characterization of
mannitol-negative methicillin-resistant *Staphylococcus
aureus*. Diagn Microbiol Infect Dis.

[B19] Swenson JM, Tenover FC, the Cefoxitin Disk Study Group (2005). Results of Disk diffusion testing with cefoxitin correlate with
presence of *mecA* in *Staphylococcus
aureus*. J Clin Microbiol.

[B20] Tsubakishita S, Kuwahara-Arai K, Sasaki T (2010). Origin and molecular evolution of the determinant of methicillin
resistance in staphylococci. Antimicrob Agents Chemother.

[B21] Zhang K, McClure JA, Elsayed S (2005). Novel multiplex PCR assay for characterization and concomitant
subtyping of staphylococcal cassette chromosome *mec* types I
to V in methicillin-resistant *Staphylococcus
aureus*. J Clin Microbiol.

